# Effectiveness of low-frequency, non-navigated repetitive transcranial magnetic stimulation for obsessive-compulsive disorder: a systematic review and meta-analysis of randomized controlled trials

**DOI:** 10.3389/fpsyt.2026.1887581

**Published:** 2026-07-17

**Authors:** Xiaojiang Liu, Jiayi Li, Jun Li

**Affiliations:** 1 Neurosurgery, Neurosurgery of Hai'an People’s Hospital, Nantong, China; 2 Medical Department, School of Medicine, Xinglin College, Nantong University, Nantong, China

**Keywords:** meta-analysis, neuromodulation, obsessive-compulsive disorder, randomized controlled trial, repetitive transcranial magnetic stimulation

## Abstract

**Background:**

Obsessive-compulsive disorder (OCD) imposes a substantial burden on patients and health systems alike: despite established pharmacological and psychological interventions, up to 40% of patients fail to achieve full remission with first-line treatment, driving persistent interest in alternative neuromodulation approaches. Among the neuromodulation strategies under investigation, low-frequency (predominantly 1 Hz), non-neuronavigated repetitive transcranial magnetic stimulation (rTMS) represents a surface-coil protocol class distinct from the FDA-cleared deep TMS approach for OCD, yet its effectiveness remains uncertain.

**Objective:**

To estimate the pooled effect of low-frequency (predominantly 1 Hz), non-neuronavigated rTMS versus sham stimulation on Yale-Brown Obsessive-Compulsive Scale (Y-BOCS) scores, and to determine whether stimulation target, treatment duration, or frequency systematically moderates that effect within this protocol class.

**Methods:**

This PRISMA 2020-compliant systematic review and meta-analysis (PROSPERO: CRD420261302647) searched PubMed, Embase, and Web of Science from inception through May 2025. Eligible studies were sham-controlled RCTs reporting Y-BOCS total scores; risk of bias was assessed using the Cochrane RoB 2.0 tool. The primary outcome was the between-group mean difference (MD) in Y-BOCS scores under a DerSimonian-Laird random-effects model, selected per pre-specified criteria (random effects if I² ≥50% or Q-test P ≤ 0.1). Pre-specified subgroup analyses examined stimulation target, treatment duration, and frequency.

**Results:**

Fourteen RCTs (N = 460; 252 active rTMS, 208 sham or control) were included. The primary pooled analysis yielded a mean difference (MD) in Y-BOCS scores of −0.69 (95% CI: −1.75 to 0.38; P = 0.21; I² = 58%), indicating no statistically significant benefit of rTMS over sham. Moderate heterogeneity was substantially reduced by exclusion of a single outlying trial, without altering the null direction of the estimate. All pre-specified subgroup analyses — by stimulation target (DLPFC vs. SMA), treatment duration (2–12 weeks), and stimulation frequency (1 Hz vs. 10 Hz) — were non-significant, and leave-one-out sensitivity analysis confirmed robustness across all 14 permutations.

**Conclusion:**

Pooled evidence from 14 RCTs does not support a statistically significant benefit of low-frequency, non-neuronavigated rTMS over sham for OCD symptom reduction (MD = −0.69, 95% CI: −1.75 to 0.38; P = 0.21). This finding is specific to low-frequency, non-neuronavigated surface-coil protocols and should not be interpreted as evidence against rTMS efficacy more broadly; approved deep TMS and neuronavigated protocols were outside the scope of this review. These results indicate that this low-frequency, non-navigated protocol class lacks demonstrated efficacy in controlled trials. Whether neuronavigation and cortical field verification can bridge this performance gap requires adequately powered trials designed for direct protocol comparison.

## 1 Introduction

Obsessive-compulsive disorder (OCD) is a chronic and disabling psychiatric condition in which persistent intrusive thoughts and ritualistic behaviors impose substantial distress, functional impairment, and social burden (1). Patients typically retain insight into the irrationality of their symptoms yet remain unable to suppress them — a discrepancy that distinguishes OCD from many other anxiety-spectrum disorders and poses a substantial challenge to treatment. Although serotonin reuptake inhibitors and cognitive-behavioral therapy constitute established first-line interventions, up to 40% of patients fail to achieve adequate remission, and a further proportion discontinues treatment owing to intolerable side effects or insufficient efficacy (1, 2). For this treatment-resistant subgroup, the search for effective neuromodulation-based alternatives has become a priority in psychiatric research.

Among the neuromodulation strategies explored for treatment-resistant OCD — including deep brain stimulation (DBS), vagus nerve stimulation (VNS), transcranial direct current stimulation (tDCS), and electroconvulsive therapy (ECT) (3) — rTMS occupies a distinctive position by virtue of its non-invasive profile. It requires neither anesthesia nor surgical intervention and can be administered in standard outpatient settings, a practical advantage that more invasive alternatives cannot offer. Mechanistically, rTMS delivers focal electrical currents to targeted cortical regions via rapidly alternating magnetic pulses, modulating excitability in a frequency-dependent manner: low-frequency stimulation (typically 1 Hz) is generally inhibitory, whereas higher frequencies produce facilitatory effects (4, 5). Having received regulatory approval for treatment-resistant depression and, subsequently, for OCD via the deep TMS H7-coil protocol, rTMS has attracted growing scientific and clinical interest — particularly given the well-characterized dysfunction of cortico-striato-thalamo-cortical (CSTC) circuits that underlies OCD pathophysiology (3, 6). The present review focuses exclusively on surface-coil, non-neuronavigated protocols and does not evaluate the approved H7-coil paradigm.

Despite this promise, the evidence base for rTMS in OCD remains heterogeneous and difficult to interpret in aggregate. Individual RCTs have yielded contradictory results, a pattern that the meta-analytic literature has not fully resolved. Fitzsimmons et al. (2022) identified significant benefits for select protocols in a pairwise and network meta-analysis (7); Steuber and McGuire (2023) reported a pooled effect of Hedges’ g = 0.65 across 28 RCTs, with inhibitory stimulation over the right dorsolateral prefrontal cortex (DLPFC) and supplementary motor area (SMA) showing the strongest evidence (8). Network meta-analyses by Vinod et al. (2024) (9), Perera et al. (2021) (10), and Liang et al. (2021) (11) have similarly identified protocol-dependent variation in effect magnitude across rTMS paradigms. Tseng et al. (2025) extended this picture by evaluating 24 distinct non-invasive brain stimulation approaches and demonstrating that efficacy varied substantially across methods and targets (12), while Dehghani-Arani et al. (2024) introduced an important mechanistic dimension: in their analysis of 27 RCTs, the magnitude of rTMS-induced electrical fields in targeted circuits — not merely the nominal stimulation site — emerged as a critical predictor of clinical response (13). What these analyses share, however, is a tendency to pool trials across widely differing levels of protocol sophistication, which may obscure whether unguided, low-frequency surface-coil stimulation — a protocol class distinct from FDA-cleared deep TMS approaches — is itself effective.

A clinically consequential question therefore remains inadequately addressed: does this low-frequency (predominantly 1 Hz), non-neuronavigated protocol class — which may still be used off-label in some centers — produce statistically significant reductions in OCD severity when evaluated against sham in controlled trials? Critically, this question differs from asking whether rTMS broadly is effective for OCD: it targets this specific protocol class and its evidence base, not the field as a whole. The present systematic review and meta-analysis was designed to answer this question directly, with two pre-specified objectives: to estimate the pooled effect of this protocol class versus sham on Y-BOCS scores, and to determine whether any protocol-level parameter — stimulation target, treatment duration, or stimulation frequency — systematically accounts for variability in that effect.

## 2 Methods

### 2.1 Search strategy and registration

This systematic review and meta-analysis was conducted in accordance with the PRISMA 2020 guidelines and prospectively registered in PROSPERO (registration number: CRD420261302647). A comprehensive electronic search of PubMed, Embase, and Web of Science was performed from database inception through May 2025, combining MeSH terms with free-text keywords to maximize retrieval sensitivity.

The PubMed search formula was: (obsessive-compulsive disorder[MeSH Terms] OR obsessive-compulsive disorder[Title/Abstract] OR obsessive compulsive disorder[Title/Abstract] OR OCD[Title/Abstract] OR obsessive compulsive[Title/Abstract]) AND (transcranial magnetic stimulation[MeSH Terms] OR transcranial magnetic stimulation[Title/Abstract] OR repetitive transcranial magnetic stimulation[Title/Abstract] OR rTMS[Title/Abstract] OR RTMS[Title/Abstract] OR repetitive TMS[Title/Abstract]). Filter: Randomized Controlled Trial. Reference lists of included studies and relevant reviews were also hand-searched.

### 2.2 Eligibility criteria

Eligibility criteria were defined prospectively using the PICOS framework: Population — adults or adolescents with a confirmed OCD diagnosis; Intervention — rTMS via any surface-coil, non-H7-coil protocol; Comparator — sham stimulation; Outcome — Y-BOCS total score; Study design — randomized controlled trial. Studies were included if they (1): used an RCT design (2); enrolled participants meeting DSM-5 (14) or ICD-11 (15) criteria for OCD with no restriction on age, sex, or illness duration (3); administered rTMS in the experimental arm (4); used sham stimulation as the primary comparator — studies employing pharmacological treatment or waitlist control as the sole comparator were excluded, whereas studies that included both sham and an additional active or waitlist arm were eligible, with only the rTMS-versus-sham contrast entered into the quantitative synthesis; and (5) reported total Y-BOCS score as the primary outcome measure. Non-RCTs, animal studies, conference abstracts, case reports, letters, reviews, and studies with unavailable or unextractable data were excluded. Studies comparing two active stimulation protocols without a sham or placebo control arm were also excluded, as they do not permit estimation of the effect of rTMS relative to no active treatment. Trials employing the FDA-cleared deep TMS H7-coil protocol (targeting the medial prefrontal cortex and anterior cingulate cortex) were identified during full-text screening but excluded because this device-specific, regulatory-approved paradigm constitutes a qualitatively distinct intervention from the surface-coil, non-neuronavigated low-frequency protocols that constitute the focus of this review. Including these trials would have introduced protocol heterogeneity inconsistent with the specific clinical question under investigation.

### 2.3 Data extraction and risk of bias

Two reviewers (X. Liu and J. Li [Jiayi Li]) independently screened titles, abstracts, and full texts; disagreements were resolved by discussion or, where consensus could not be reached, by arbitration with the senior author (J. Li [Jun Li]). For each eligible trial, the following data were independently extracted: first author, publication year, stimulation target, stimulation frequency, treatment duration, sample size, and Y-BOCS total scores at baseline and post-treatment, together with any reported adverse events. Discrepancies in data extraction were reconciled through re-examination of source documents. Risk of bias was assessed independently by both reviewers using the Cochrane RoB 2.0 tool across five domains (1): randomization process (2); deviations from intended interventions (3); missing outcome data (4); measurement of the outcome; and (5) selection of the reported result. Each trial received an overall rating of low risk, some concerns, or high risk.

### 2.4 Statistical analysis

Analyses were performed using Review Manager v5.3. The primary outcome was the post-treatment endpoint mean difference (MD) in Y-BOCS total scores between rTMS and sham arms. All 14 included trials reported post-treatment absolute scores; change-from-baseline pooling was therefore not required and not performed, as endpoint values were uniformly available and directly comparable across studies. The mean difference (MD) was preferred over standardized mean difference (SMD) because all studies employed the same outcome instrument (Y-BOCS), rendering raw score units directly interpretable. A clinically meaningful change in Y-BOCS is generally considered to require a reduction of at least 4 to 6 points based on expert consensus and prior trial data, providing a contextual benchmark for interpreting the pooled estimate. Continuous outcomes were summarized as MD with 95% confidence intervals (CI). Heterogeneity was assessed using I² and Q statistics; a DerSimonian-Laird random-effects model was applied when I² ≥50% or Q-test P ≤ 0.1; otherwise a fixed-effects model was used. Pre-specified subgroup analyses examined stimulation target (DLPFC vs. SMA), treatment duration, and frequency (1 Hz vs. 10 Hz); subgroup analyses based on five or fewer studies were pre-designated as exploratory. The robustness of the primary result was evaluated using leave-one-out sensitivity analysis, in which each trial was systematically excluded and the pooled estimate recalculated. Publication bias was assessed by funnel plot and Egger’s test; asymmetry was addressed with the trim-and-fill method using Stata v15.0.

## 3 Results

### 3.1 Study selection

**Figure 1:**
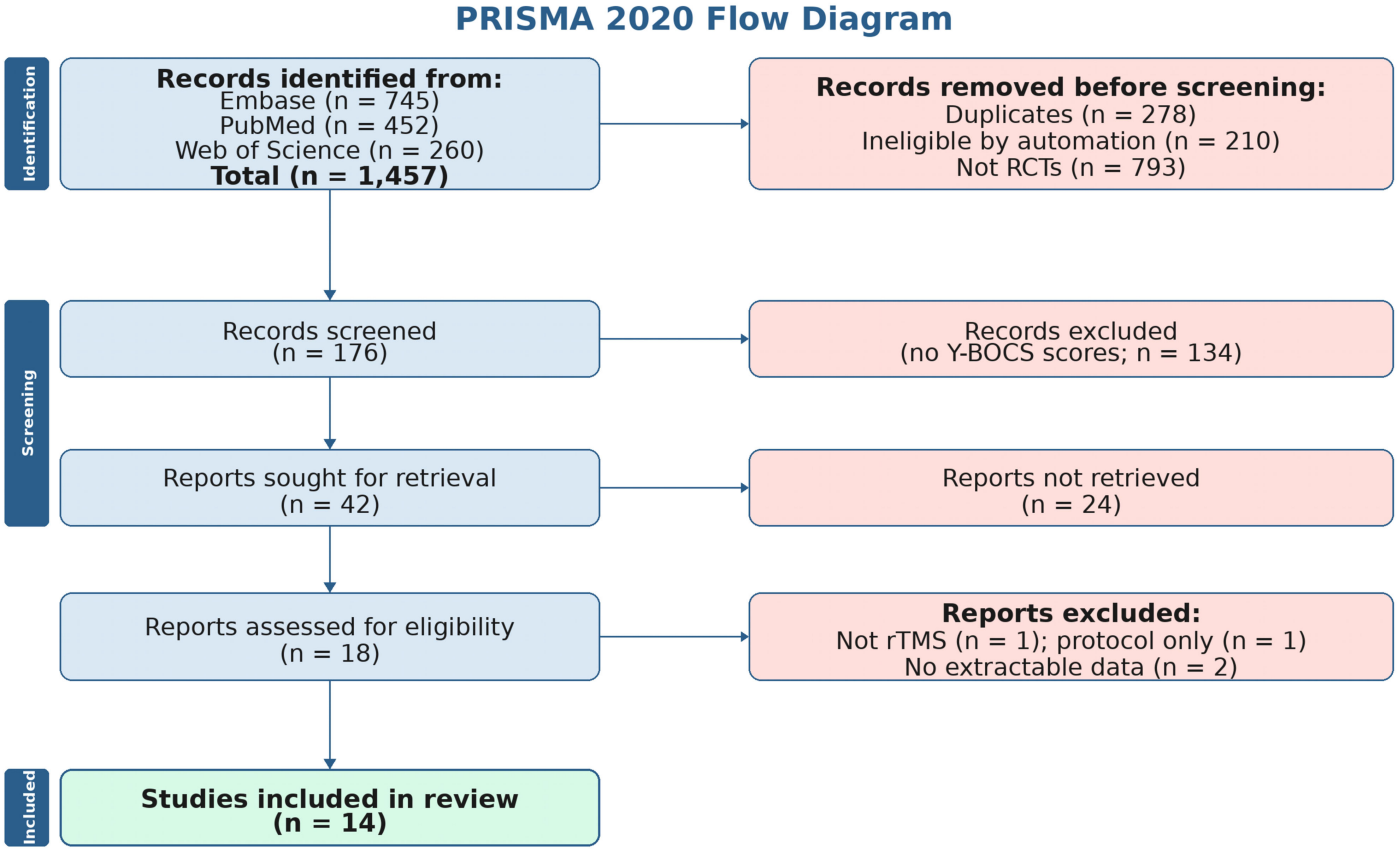
PRISMA 2020 flow diagram showing study identification and selection.

The combined database search retrieved 1, 457 records. After automated deduplication and sequential screening of titles, abstracts, and full texts, 14 RCTs satisfied all eligibility criteria and were carried forward to quantitative synthesis (**Figure 1**). These trials enrolled 460 participants in total — 252 allocated to active rTMS and 208 to sham or control conditions — with treatment durations ranging from 2 to 12 weeks and stimulation targets distributed between the DLPFC (five trials) and the SMA (nine trials). A notable feature of this evidence base is its near-uniform reliance on 1 Hz stimulation: 13 of the 14 included trials employed this inhibitory protocol. Although this review was designed to address low-frequency rTMS broadly, the dataset is de facto a 1 Hz literature. One trial used 10 Hz (Sarkhel et al., 2010) (16); this trial was retained to avoid *post-hoc* exclusion, but results are reported separately and no conclusions about 10 Hz rTMS can be drawn from a single underpowered comparison. The term “low-frequency rTMS” throughout this manuscript refers primarily to the 1 Hz protocols that constitute 93% of the included evidence.

### 3.2 Risk of bias

**Figure 2:**
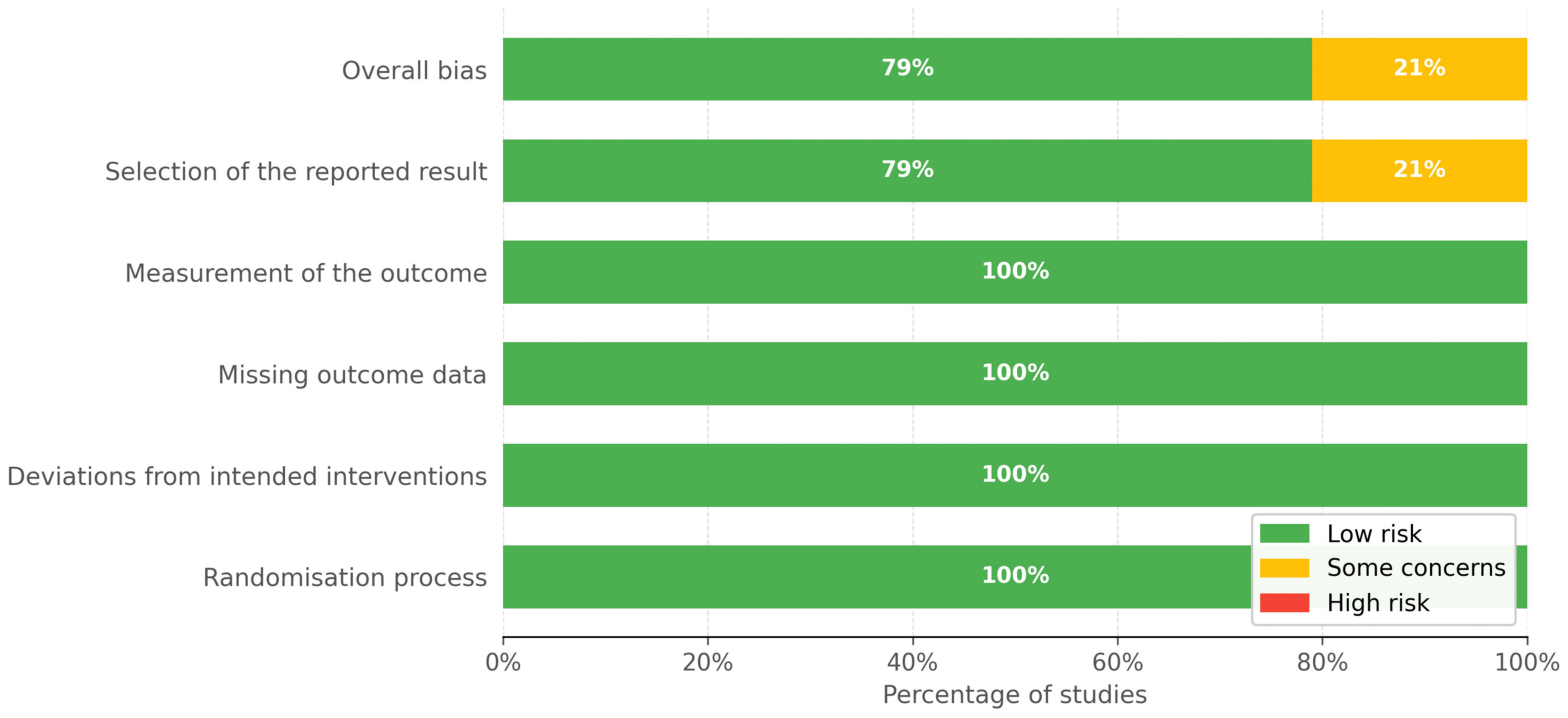
Risk of bias summary (RoB 2.0): percentage distribution across domains.

**Figure 3:**
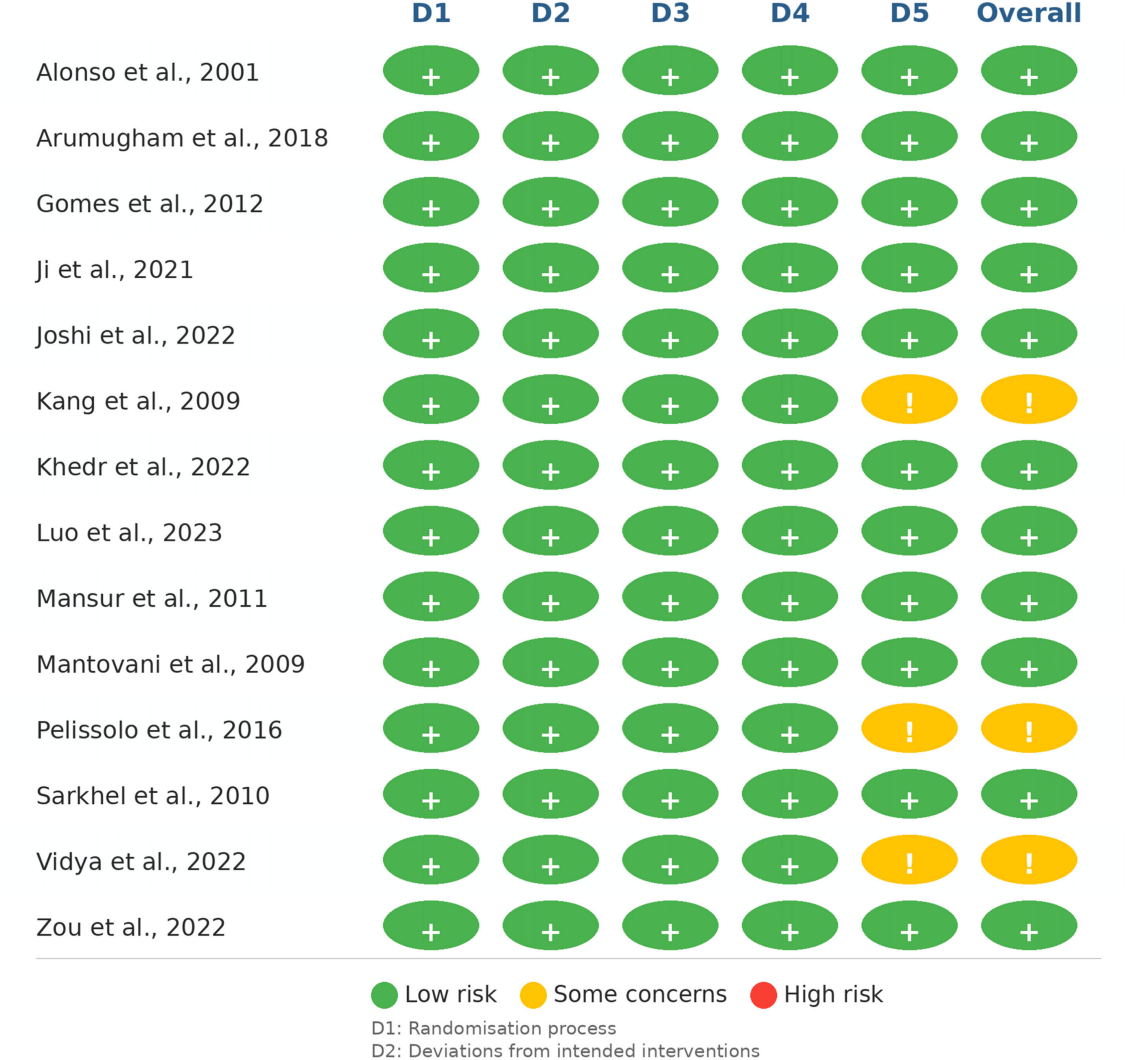
Risk of bias assessment by study (RoB 2.0 traffic light plot). Green, low risk; yellow, some concerns.

The methodological quality of the included literature was satisfactory. Eleven of 14 trials were rated as low risk across all five RoB 2.0 domains (16–25); the three remaining studies — Vidya et al. (2022) (26), Pelissolo et al. (2016) (27), and Kang et al. (2009) (28) — received “some concerns” ratings confined to Domain 5 (selection of the reported result), where unexpectedly favorable control-arm outcomes raised the possibility of selective reporting or protocol deviations. This domain-specific pattern — with concerns absent from randomization, allocation concealment, missing data, and outcome measurement — indicates that 11 of 14 trials (79%) carried no identified risk of bias across any domain, while flagging the three affected trials as warranting cautious interpretation. **Figure 2** and **3** present the domain-level and study-level summaries, respectively.

### 3.3 Primary analysis

**Figure 4:**
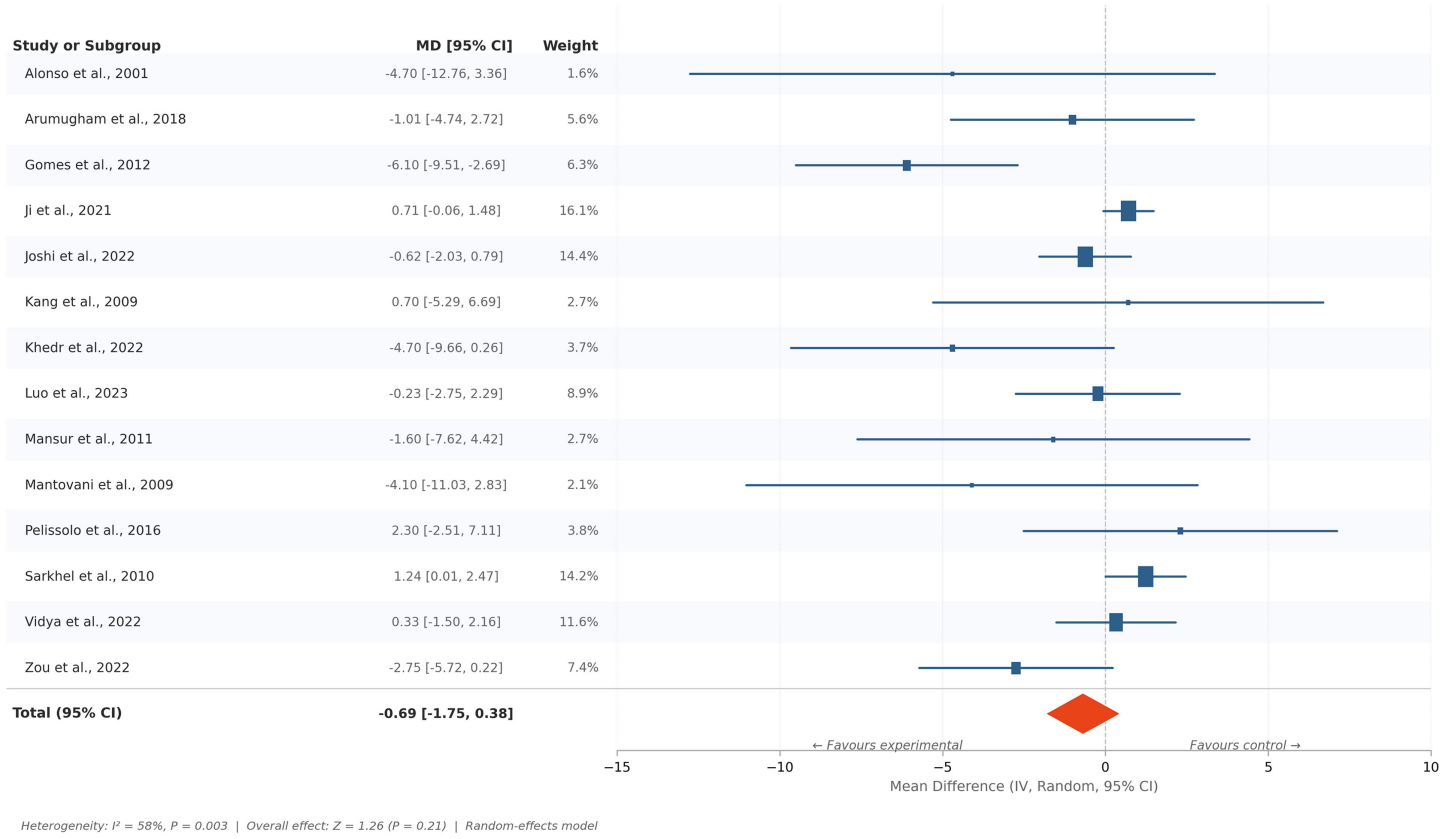
Overall forest plot: rTMS vs. sham across 14 randomized controlled trials. MD, mean difference; CI, confidence interval.

The primary pooled analysis (**Figure 4**) yielded a mean difference in Y-BOCS scores of −0.69 (95% CI: −1.75 to 0.38; P = 0.21; I² = 58%), indicating no statistically significant benefit of low-frequency rTMS over sham. The 95% confidence interval (−1.75 to 0.38) crosses zero; the upper bound of 0.38 Y-BOCS points falls well below the 4 to 6-point threshold generally considered clinically meaningful, providing no indication of a clinically important treatment effect. Between-study heterogeneity was moderate (Cochran’s Q = 30.98, df = 13, P = 0.003); sensitivity analysis identified a single high-baseline-severity outlier (29) as the primary source, with heterogeneity reducing to near zero upon its exclusion (see Section 3.5 and Discussion).

### 3.4 Subgroup analyses

**Figure 5:**
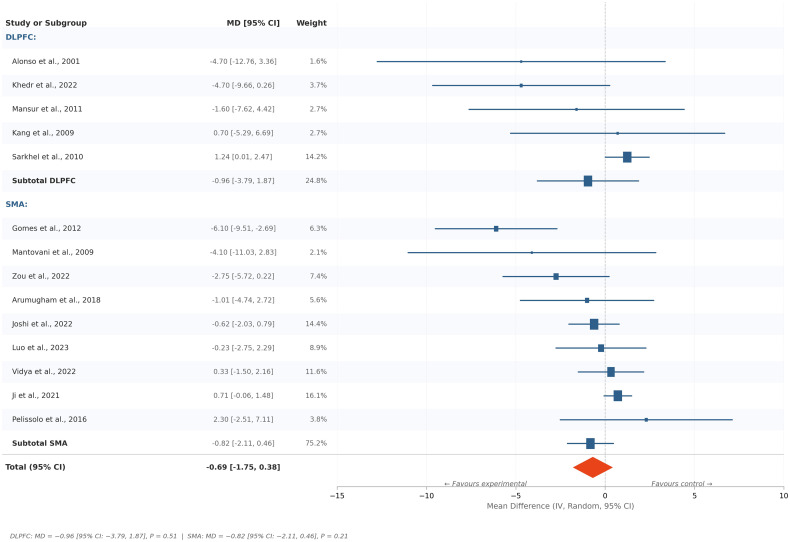
Forest plot by stimulation target: DLPFC vs. supplementary motor area (SMA) subgroups.

Stratification by stimulation target revealed no differential treatment effect (**Figure 5**). Trials targeting the DLPFC (n = 5) yielded a pooled MD of −0.96 (95% CI: −3.79 to 1.87; P = 0.51); those directed at the SMA (n = 9) produced MD = −0.82 (95% CI: −2.11 to 0.46; P = 0.21). Neither estimate reached statistical significance, and the between-subgroup contrast was non-significant (P = 0.93). The wide confidence interval for the DLPFC subgroup — spanning nearly 5.7 Y-BOCS points — reflects the small number of contributing trials. This subgroup analysis should be regarded as exploratory and underpowered; the absence of a significant subgroup effect should not be interpreted as evidence that stimulation target is unimportant.

**Figure 6:**
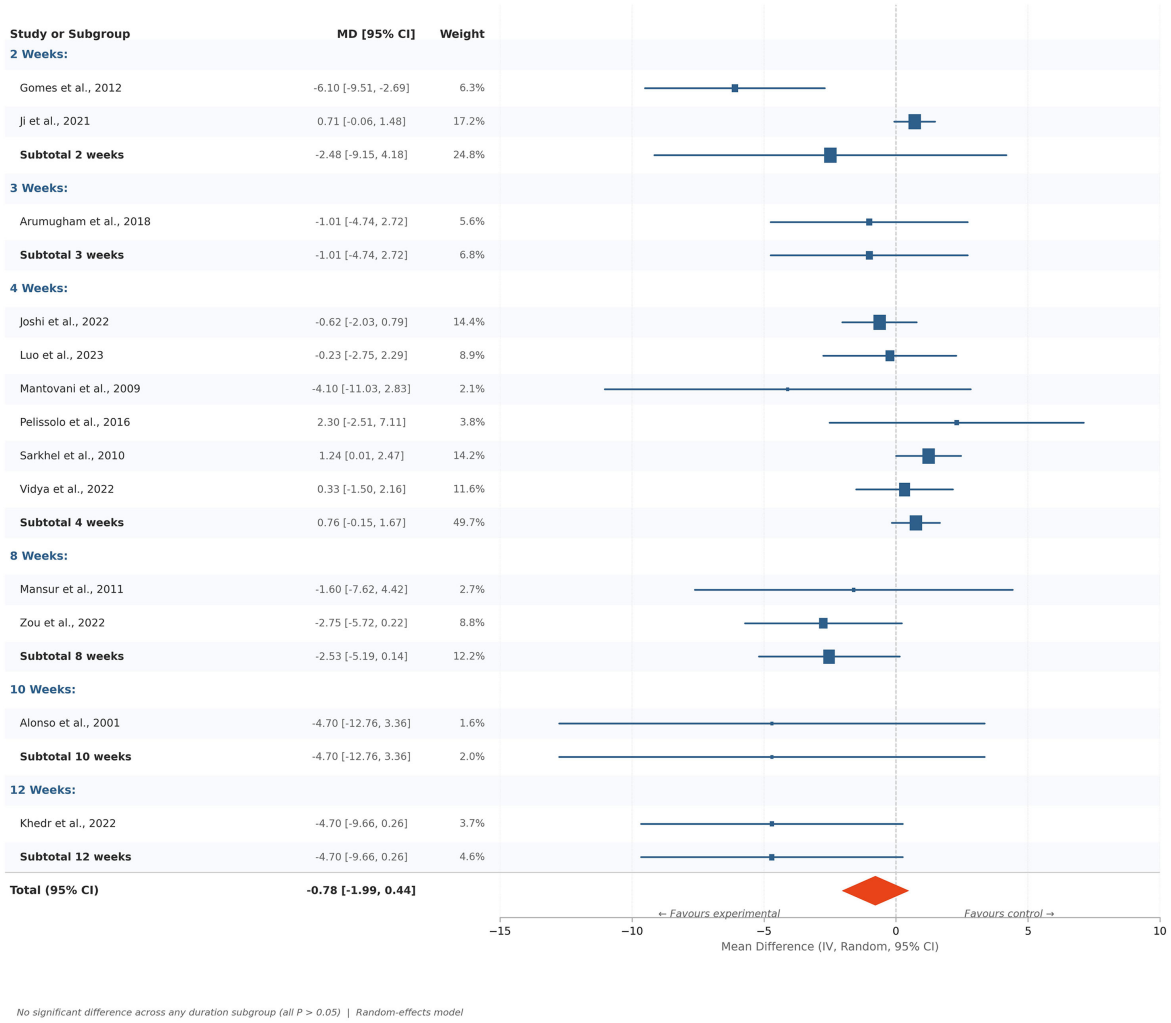
Forest plot by treatment duration subgroups. All P-values > 0.05.

Treatment duration was equally uninformative as an explanatory variable (**Figure 6**). The four duration categories represented in this dataset — 2 weeks (MD = −2.48; P = 0.46), 3 weeks (MD = −1.01; P = 0.60), 4 weeks (MD = 0.76; P = 0.10), and 8 weeks (MD = −2.53; P = 0.06) — yielded uniformly non-significant pooled estimates, and the test for between-subgroup differences was non-significant. The 8-week subgroup produced a P-value of 0.06, approaching but not crossing the conventional significance threshold; given that only two trials contributed to this estimate, this observation warrants caution rather than clinical inference. One trial did not report treatment duration and was excluded from this analysis (23).

**Figure 7:**
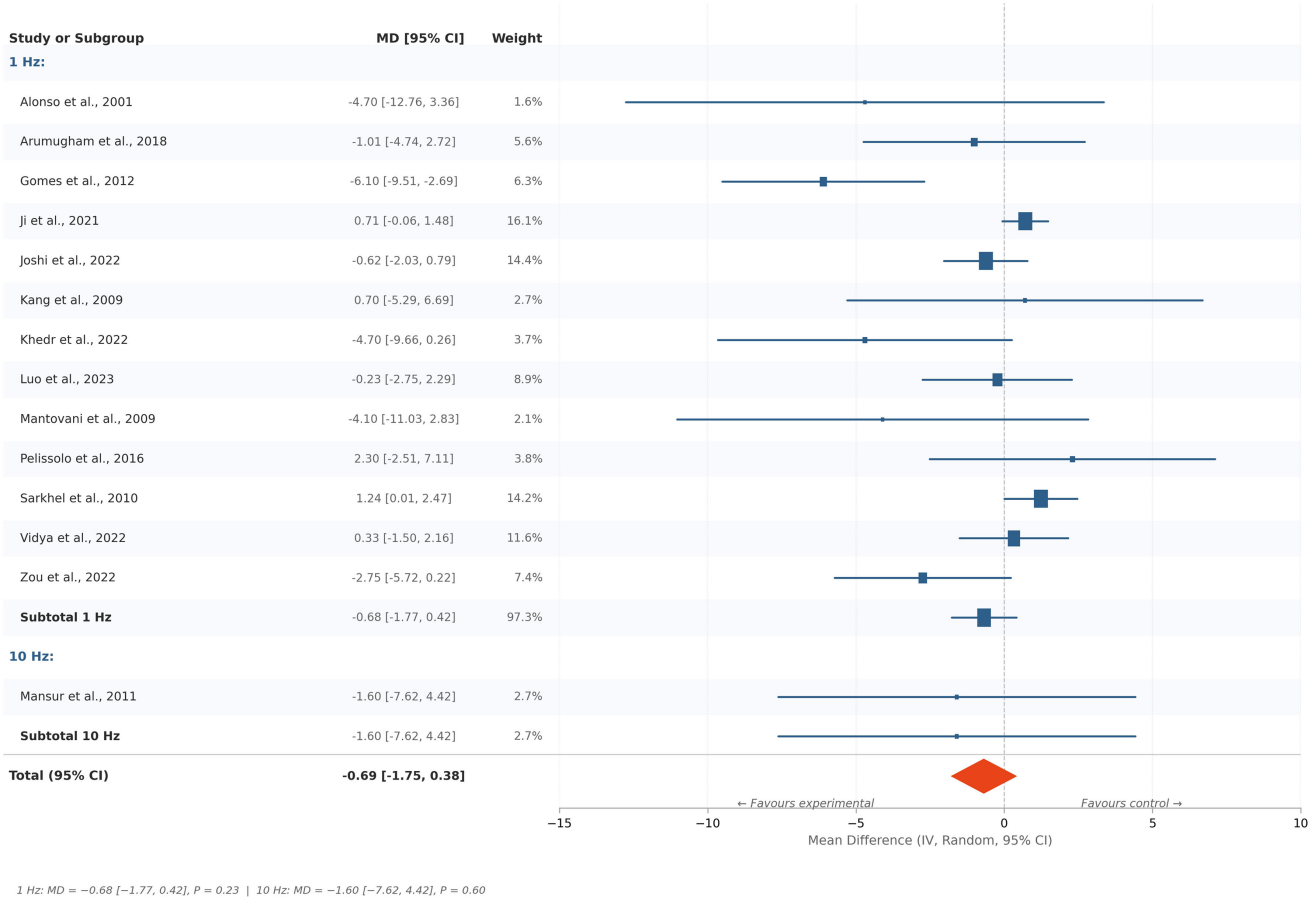
Forest plot by stimulation frequency: 1 Hz vs. 10 Hz subgroups.

The frequency subgroup analysis (**Figure 7**) produced results consistent with the preceding comparisons. The 13 trials employing 1 Hz stimulation — by far the dominant protocol in this literature — yielded a pooled MD of −0.68 (95% CI: −1.77 to 0.42; P = 0.23). The single 10 Hz trial produced MD = −1.60 (95% CI: −7.62 to 4.42; P = 0.60). This single-study subgroup is exploratory only; no conclusions about higher-frequency rTMS can be drawn from a single underpowered comparison. Neither frequency demonstrated a statistically significant effect over sham.

### 3.5 Sensitivity analysis

Leave-one-out sensitivity analysis confirmed the stability of the primary result: irrespective of which trial was excluded, the pooled P-value remained above 0.05 in all 14 iterations, and the 95% CI crossed zero in every case. The MD estimates ranged from −1.05 to −0.06 across all 14 iterations (**Table 1**, which presents each leave-one-out pooled estimate), with no single exclusion producing a directionally reversed or statistically significant estimate. The null finding is therefore unlikely to be attributable to the disproportionate influence of any individual study.

**Table 1:** Leave-one-out sensitivity analysis (DerSimonian-Laird random-effects model).

Study excluded	Pooled MD (95% CI)	Weight (%)
Alonso et al., 2001 (19)	−0.48 (−1.55, 0.59)	6.92
Arumugham et al., 2018 (20)	−0.89 (−2.08, 0.31)	5.51
Gomes et al., 2012 (29)	−1.05 (−2.25, 0.15)	5.51
Ji et al., 2021 (17)	−0.82 (−1.92, 0.28)	6.57
Joshi et al., 2022	−0.61 (−1.68, 0.46)	6.89
Kang et al., 2009 (28)	−0.68 (−1.77, 0.42)	6.58
Khedr et al., 2022 (24)	−0.77 (−1.93, 0.38)	5.90
Luo et al., 2023 (18)	−0.49 (−1.53, 0.55)	7.32
22 (22)	−0.75 (−1.85, 0.35)	6.52
Mantovani et al., 2009	−0.78 (−1.99, 0.44)	5.34
Pelissolo et al., 2016 (27)	−1.05 (−2.33, 0.23)	4.82
Sarkhel et al., 2010 (16)	−0.06 (−0.88, 0.75)	11.89
Vidya et al., 2022 (26)	−0.69 (−1.81, 0.43)	6.32
Zou et al., 2022 (25)	−0.62 (−1.69, 0.45)	6.92
Overall (DL model)	−0.69 (−1.75, 0.38)	100

Each row presents the pooled MD (95% CI) obtained after excluding the named study; the final row presents the full-sample pooled estimate. MD, mean difference; CI, confidence interval.

### 3.6 Publication bias

**Figure 8:**
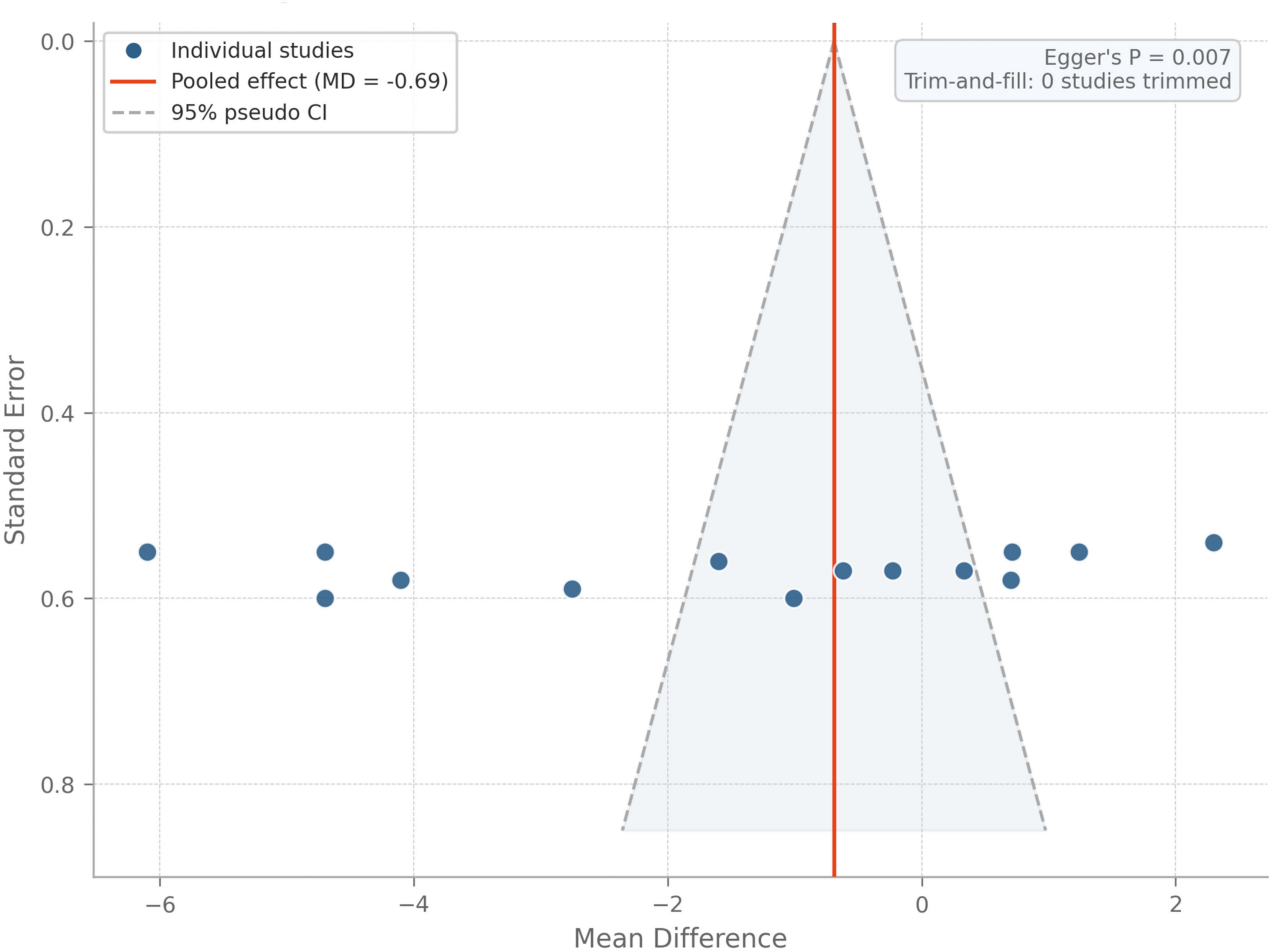
Funnel plot for publication bias assessment. Dashed lines indicate 95% pseudo-confidence interval; vertical line = pooled effect.

Funnel plot asymmetry was detected by Egger’s test (P = 0.007). Egger’s test has reduced statistical power and elevated false-positive rates with fewer than approximately 20 studies, so this result requires cautious interpretation; it nonetheless raises the possibility of small-study effects or selective publication bias. To assess whether this asymmetry materially affected the pooled estimate, trim-and-fill analysis was performed. The procedure required no studies to be imputed (number to trim = 0), and the fixed-effects pooled MD stabilized at 0.193 after the second iteration (95% CI: −0.263 to 0.650; P = 0.406) — positive in direction but non-significant, and materially different from the random-effects primary estimate owing to the high between-study variance. Under the random-effects model, the corrected estimate was MD = −0.548 (95% CI: −1.506 to 0.411; P = 0.263), also non-significant. Given the substantial between-study variance (Cochran’s Q = 30.981, P = 0.003; τ² = 1.273), the random-effects estimate is the primary inferential result; the trim-and-fill analysis provides additional assurance that any residual publication bias, if present, did not alter the null conclusion. The funnel plot is shown in **Figure 8**; full trim-and-fill output is presented in **Table 2**.

**Table 2:** Trim-and-fill publication bias analysis.

Analysis phase	Model	Pooled MD (95% CI)	z	P	n	Trim/fill	Q (df=13)	I² (%)
Primary	Fixed effect	0.193 (−0.263, 0.650)	0.831	0.406	14	—	30.981 (0.003)	58
Primary	Random effect	−0.548 (−1.506, 0.411)	−1.12	0.263	14	—	30.981 (0.003)	58
Trim-and-fill	Fixed effect	0.193 (−0.263, 0.650)	0.831	0.406	14	0/0	30.981 (0.003)	58
Trim-and-fill	Random effect	−0.548 (−1.506, 0.411)	−1.12	0.263	14	0/0	30.981 (0.003)	58

## 4 Discussion

The primary finding of this meta-analysis is a consistent null result: low-frequency rTMS did not produce a statistically significant reduction in Y-BOCS scores across 14 RCTs and 460 patients, and this finding held across every pre-specified subgroup — stimulation target, treatment duration, and frequency — as well as all 14 iterations of the leave-one-out sensitivity analysis. The consistency of the null result across protocol parameters strengthens the inference: it cannot be attributed to the choice of cortical target, the duration of treatment, or the disproportionate influence of any individual outlying trial. Taken together, these features indicate that the absence of a detectable effect reflects a systematic property of the low-frequency, non-navigated rTMS protocols examined here, rather than sampling variability or analytical choice.

The moderate between-study heterogeneity (I² = 58%) warrants methodological comment. Its near-complete resolution upon exclusion of Gomes et al. (2012) (29) — a trial whose participants had mean baseline Y-BOCS scores substantially higher than those of every other included study — confirms that this variance reflects population-sampling differences rather than genuine inconsistency in the treatment effect itself. This distinction matters methodologically: the null pooled estimate does not arise from opposing directional effects cancelling across trials, but reflects a stable finding within a clinically coherent population — one that excludes patients with unusually high baseline severity. A secondary implication concerns subgroup responsiveness: without participant-level data stratified by baseline severity, it remains unknown whether treatment response varies systematically with OCD symptom burden at entry — a question with direct relevance to patient selection in future trials.

The present null result is not inconsistent with the broader meta-analytic literature once protocol differences are taken into account. Pooled analyses reporting positive outcomes — including the pairwise and network meta-analysis by Fitzsimmons et al. (2022), which identified significant benefits for select protocols across multiple targets (7), and the analysis by Steuber and McGuire (2023) reporting Hedges’ g = 0.65 across 28 RCTs with inhibitory stimulation over the DLPFC and SMA showing the strongest evidence (8) — typically include trials employing neuronavigated target localization, bilateral stimulation configurations, or theta-burst paradigms that were absent from the present dataset. The protocol-dependence of efficacy was made explicit by Tseng et al. (2025), whose network meta-analysis spanning 24 non-invasive brain stimulation approaches demonstrated that outcomes differed substantially by modality and target (12). Dehghani-Arani et al. (2024) added a parameter-level dimension to this inquiry: across 27 RCTs, the magnitude of rTMS-induced electrical fields at the cortical target — rather than the nominal anatomical site — emerged as the strongest predictor of treatment response, with protocols generating larger fields consistently associated with larger effects (13). Intensity and scheduling factors are relevant even within the low-frequency paradigm: accelerated protocols using multiple daily sessions produced significant OCD symptom reductions within one to three weeks, according to the first systematic meta-analysis specifically examining this approach (30). Taken together, this body of evidence contextualizes the present null result within a framework of protocol-specificity. The hypothesis that emerges is that unguided, low-frequency stimulation may be insufficient to achieve the degree of cortical engagement associated with therapeutic efficacy in more optimized protocols — though this hypothesis was not directly tested in the present analysis and requires prospective experimental evaluation.

The practical implication is direct: clinicians should not assume that effect sizes reported in protocol-optimized research trials will translate to the unguided 1 Hz approach evaluated here. The present findings do not argue that rTMS is inherently ineffective in OCD; they indicate, more precisely, that the specific protocol class examined here — low-frequency, non-neuronavigated surface-coil stimulation — has not demonstrated statistically significant or clinically meaningful efficacy in the available controlled trial evidence. Whether the mechanism involves insufficient cortical electric field magnitude, suboptimal target localization, inadequate treatment intensity, or some combination thereof cannot be resolved from the present data and awaits prospective, mechanistically-designed trials.

Several limitations warrant acknowledgment. Regarding statistical power and internal validity: with 14 trials enrolling 460 participants, the evidence base is modestly sized and may lack power to detect small but clinically meaningful effects. Residual heterogeneity after excluding the Gomes et al. (2012) outlier (29) reflects unmodelled clinical covariates — variation in concomitant medication, OCD subtype, illness duration, and baseline severity — that aggregate-level analysis cannot resolve. Subgroup analyses for the DLPFC target (n = 5) and 10 Hz frequency (n = 1) rested on too few contributing trials to support reliable inference; these should be treated as hypothesis-generating only, and their non-significant results must not be interpreted as evidence against the relevance of target or frequency as treatment moderators. Regarding generalizability and evidence overlap: the trials analyzed here likely overlap to some degree with those in prior meta-analyses by Berlim et al. (2), Fitzsimmons et al. (7), and Steuber and McGuire (8) — formal overlap analyses were not conducted, so the precise extent of shared studies cannot be quantified — and the incremental contribution of the present work therefore lies specifically in its exclusive focus on the low-frequency, non-navigated protocol class rather than pooling across heterogeneous protocol types. Although the trim-and-fill analysis identified no studies requiring imputation, funnel plot asymmetry (Egger’s P = 0.007) means that small-study effects or selective non-publication of negative trials cannot be excluded as contributors to the observed pattern. The search was restricted to PubMed, Embase, and Web of Science; a comprehensive review would ideally also include the Cochrane Central Register of Controlled Trials (CENTRAL), clinical trial registries, and grey literature. This omission may have introduced retrieval bias and precluded identification of unpublished negative trials. Regarding recent evidence: the search was conducted through May 2025, while this manuscript was submitted in 2026. Given the rapid evolution of the rTMS-OCD literature, relevant trials may have been published after the search cutoff. Two RCTs identified close to the cutoff were excluded for methodological reasons — Fitzsimmons et al. (2025) employed low-intensity vertex rTMS as the control condition rather than inert sham (31), and Gautam et al. (2025) compared two active protocols without a sham arm (32). Both are cited as contextual evidence above.

These limitations notwithstanding, the findings carry clear implications for research design. The most pressing need is not for additional trials of unguided low-frequency protocols, but for adequately powered, pre-registered RCTs that directly compare neuronavigation-guided, cortical-field-verified protocols against the unguided approach evaluated here. The methodological frameworks proposed by Fitzsimmons et al. (2025) and the ongoing MAGNITUDE trial protocol (33) — both integrating individualized fMRI-based target localization alongside active control conditions — illustrate the design features such future trials should incorporate, though the MAGNITUDE trial’s results are not yet available and its protocol has not itself been validated by completed outcomes. Beyond protocol refinement, combination strategies pairing rTMS with exposure and response prevention (ERP) merit systematic investigation, as stimulation-induced neuroplasticity may potentiate extinction learning during concurrent ERP. Whether accelerated protocols, with their compressed treatment timelines and higher cumulative pulse densities, confer benefits beyond those of standard-duration approaches in OCD specifically also warrants rigorous evaluation. These questions, which cannot be resolved through aggregate synthesis of existing trial data alone, define the empirical agenda for the field.

## 5 Conclusion

Across 14 randomized controlled trials and 460 patients, low-frequency, non-neuronavigated rTMS did not produce a statistically significant reduction in OCD symptom severity compared with sham (MD = −0.69, 95% CI: −1.75 to 0.38; P = 0.21), a finding that remained consistent across all pre-specified subgroup analyses and leave-one-out sensitivity tests. This conclusion is specific to the low-frequency, non-navigated protocol class examined here and should not be interpreted as a general statement about rTMS for OCD: the approved deep TMS H7-coil paradigm and neuronavigated surface-coil protocols, which were outside the scope of this review, have been examined in separate meta-analyses with more favorable results (7, 8). What the present evidence indicates is that this low-frequency, non-navigated approach lacks demonstrated efficacy in the available controlled trial record. Whether neuronavigation, verified cortical field delivery, and accelerated stimulation schedules can translate the mechanistic promise of rTMS into reproducible clinical benefits in an unselected OCD population is a question that only directly comparative, adequately powered trials — designed explicitly to test this translational gap — can answer.

## Data Availability

The original contributions presented in the study are included in the article/supplementary material. Further inquiries can be directed to the corresponding author.
